# Giant Cystic Pheochromocytoma Associated With Neurofibromatosis Type 1: A Case Report

**DOI:** 10.7759/cureus.60151

**Published:** 2024-05-12

**Authors:** Zineb Serhane, Sara Hassane, Hayat Aynaou, Houda Salhi, Hanan Elouahabi

**Affiliations:** 1 Department of Endocrinology, Diabetology, Metabolic Diseases and Nutrition, Hassan II University Hospital, Fes, MAR

**Keywords:** pigmento-vascularis phacomatosis, giant pheochromocytoma, hereditary paraganglioma-pheochromocytoma syndrome, adrenal pheochromocytoma, adrenalectomy, normetanephrine, metanephrine, neurofibromatosis type 1, cystic pheochromocytoma

## Abstract

Pheochromocytomas are tumors that develop from the chromaffin cells of the adrenal medulla. More than 40% of cases of pheochromocytomas are associated with genetic conditions such as neurofibromatosis type 1 (NF1) or von Hippel-Lindau syndrome. Cystic pheochromocytomas are rare, generally asymptomatic, and thus of bigger size at the time of diagnosis. Surgical treatment is necessary to prevent cardiovascular morbidity and malignancy risk. We report the case of a 27-year-old patient admitted for further examination of a left adrenal mass that was discovered by an abdominal CT scan in the context of abdominal pain associated with hypertension evolving for three years. The clinical examination showed the presence of multiple café au lait spots, axillary and inguinal freckling with two dermal neurofibromas diagnosed clinically, as well as Lisch nodules on bilateral ophthalmic examination, thus meeting the clinical criteria for the diagnosis of NF1. The clinical laboratory investigation showed elevated urinary metanephrine and normetanephrine levels. CT scan examination showed a 10 cm left adrenal cystic mass on abdominal CT. This mass uptake of the radioligand in metaiodobenzylguanidine (MIBG) scintigraphy without secondary extra-adrenal localization allowed the diagnosis of a seemingly benign cystic pheochromocytoma to be made. The patient was put on presurgical drug preparation with volume expansion and then underwent left unilateral adrenalectomy. The histopathological study was in favor of a rather aggressive cystic pheochromocytoma with a pheochromocytoma of the adrenal gland scaled (PASS) score of 9. Blood pressure and urine catecholamines at seven days, three months, six months, and one year after surgery were normalized.

Cystic pheochromocytoma is a rare tumor with a potentially poor prognosis. It is characterized by a more insidious evolution and a larger volume at diagnosis. It should be considered a diagnosis in patients with a cystic adrenal mass or an extra-adrenal mass with fluctuating blood pressure during surgery. This case illustrates the importance of both presurgical preparation and screening for pheochromocytoma in neurofibromatosis type 1.

## Introduction

Cystic pheochromocytomas are extremely rare catecholamine-secreting neuroendocrine tumors that arise from the chromaffin cells of the adrenal medulla. This form is more voluminous than its solid counterpart and has an insidious evolution, making up about 20% of all pheochromocytomas [1,2]. It can be challenging to differentiate it from other abdominal cystic masses [3,4], which delays the diagnosis [3] and increases the risk of intraoperative hemodynamic complications during surgical manipulation.

Up to 40% of pheochromocytomas are secondary to germline mutation in one of more than twenty reported susceptibility genes. A loss-of-function mutation in one of these predisposing genes, neurofibromatosis type 1 (NF1), clinically leads to neurofibromatosis type 1 [5]. Early screening for pheochromocytoma in this population is considered to be of paramount importance in order to maximize the chances of early diagnosis and treatment; therefore reducing morbi-mortality [6].

The association between NF1 and pheochromocytoma is well-established [7]. A clinical presentation of a cystic pheochromocytoma is distinctly uncommon, and the associations thereof with specific germline variants of NF1 have not been investigated. In this article, we present the case of a young patient meeting clinical diagnostic criteria for neurofibromatosis type 1 and presenting with cystic pheochromocytoma. In addition, a brief literature review of the characteristics of cystic pheochromocytomas and their association with NF1 is presented.

## Case presentation

A 27-year-old woman, born from a first-degree consanguineous marriage, was referred to our endocrinology OPD department for assessment of an adrenal incidentaloma. The medical history of the patient revealed chronic abdominal pain, paroxysmal headaches, palpitation, and profuse sweating associated with chronic hypertension, asthenia, and weight loss. Family history revealed the existence of café au lait spots in two of her children, with no known history of hypertension or familial endocrine disorders.

**Figure 1:**
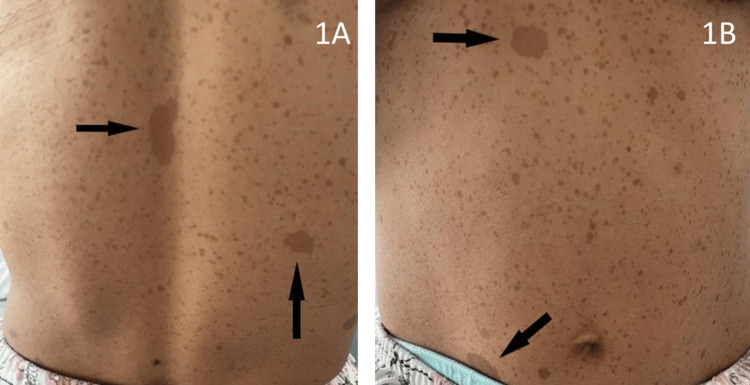
Clinical images: café au lait spots (arrows) of more than 1.5 cm in diameter and scattered freckling on the whole trunk.

**Figure 2:**
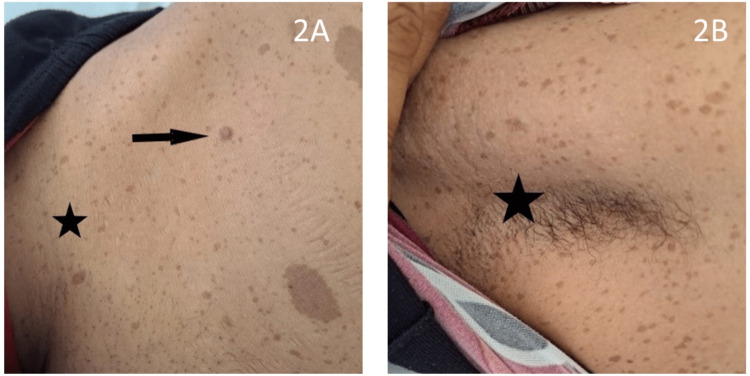
Clinical images: inguinal and axillary freckling (stars), dermal neurofibroma (arrow).

Upon examination, the patient was found to be in fairly good general condition, with a BP of 138/94 mmHg under no treatment, tachycardia at 110 bpm, and tenderness of the left flank at gentle abdominal palpation. The cutaneous examination revealed the presence of more than six cafe au lait spots greater than 1.5 cm in diameter, predominantly scattered on the trunk (Figures 1A, 1B), with axillary and inguinal freckling and two cutaneous neurofibromas (Figures 2A, 2B).

**Figure 3:**
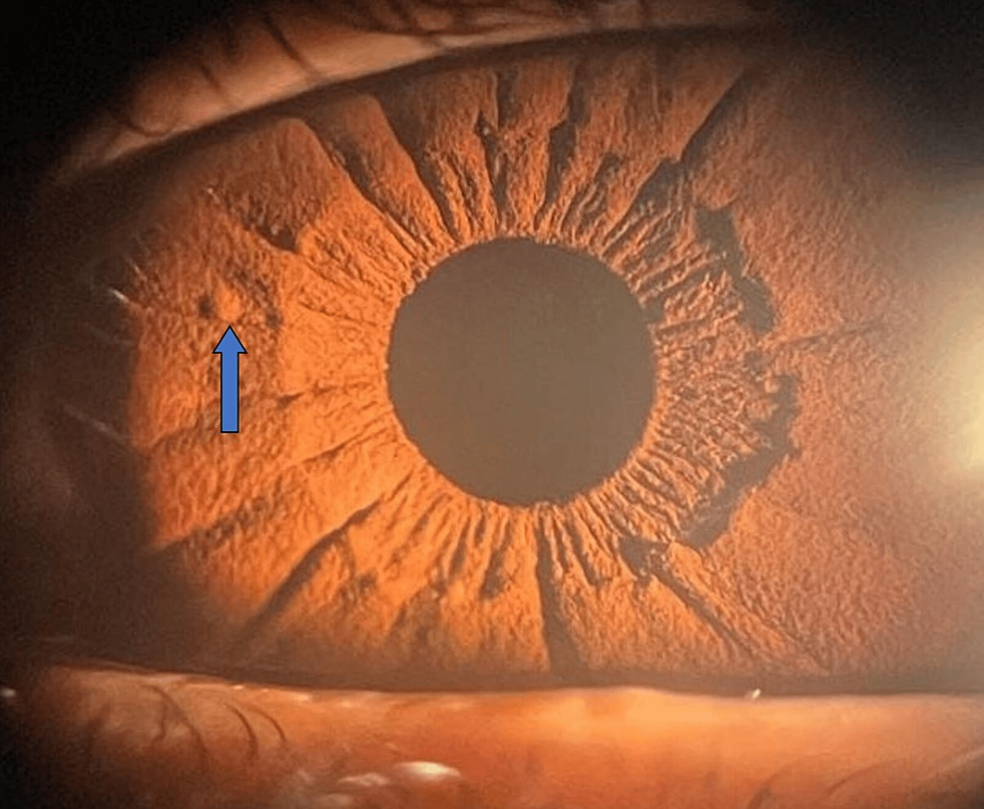
Clinical picture: ophthalmic examination showing Lisch nodule (arrow). Note: This image was kindly provided by Dr. A. Maghraoui.

A bilateral ophthalmic examination revealed the presence of several Lisch nodules (Figure 3), predominantly in the right eye. The urinary concentrations of catecholamine metabolites were elevated (normetanephrine: 659 ug/24h-12.9×upper limit of normal (ULN), metanephrine: 515 ug/24h-16.6×ULN, and 3 ortho-methyl dopa: 138 ug/24h-2.5×ULN). A non-specific biological workup was performed and is summarized in Table 1.

**Table 1:** Non-specific biological tests. HbA1C: glycated hemoglobin, HDL: high-density lipoprotein, LDL: low-density lipoprotein.

Variables	Values
Hemoglobin	12.6 g/dl
White blood cells	3870 e/mm^3^
Neutrophils	1790 e/mm^3^
Platelets	358,000 e/mm^3^
Kaliemia	4.32 mEq/l
Fasting blood glucose	0.79 g/l
HbA1C	5.6%
Total cholesterol	1.43 g/l
Triglycerides	0.64 g/l
HDL cholesterol	0.41 g/l
LDL cholesterol	0.89 g/l

**Figure 4:**
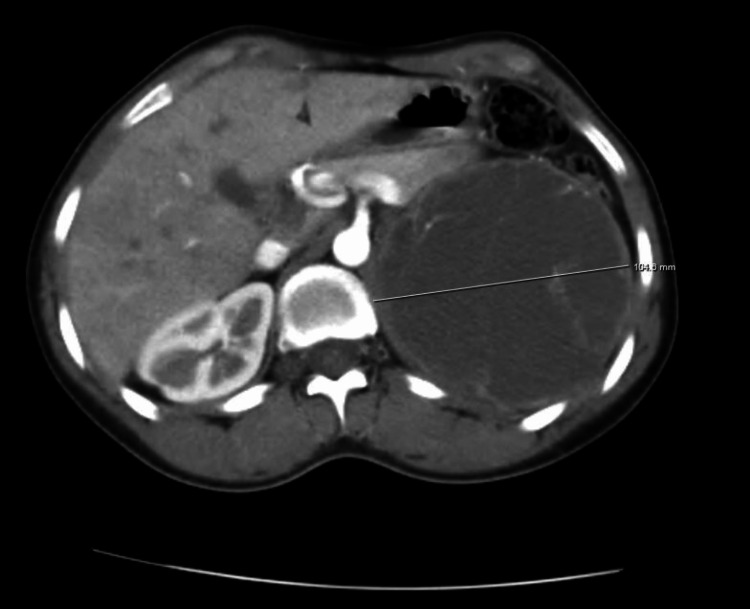
Axial section of an abdominal CT scan with IV contrast showing a 10.4 cm long cystic left adrenal mass containing fine contrast-enhanced septas and displacing the surrounding structures.

**Figure 5:**
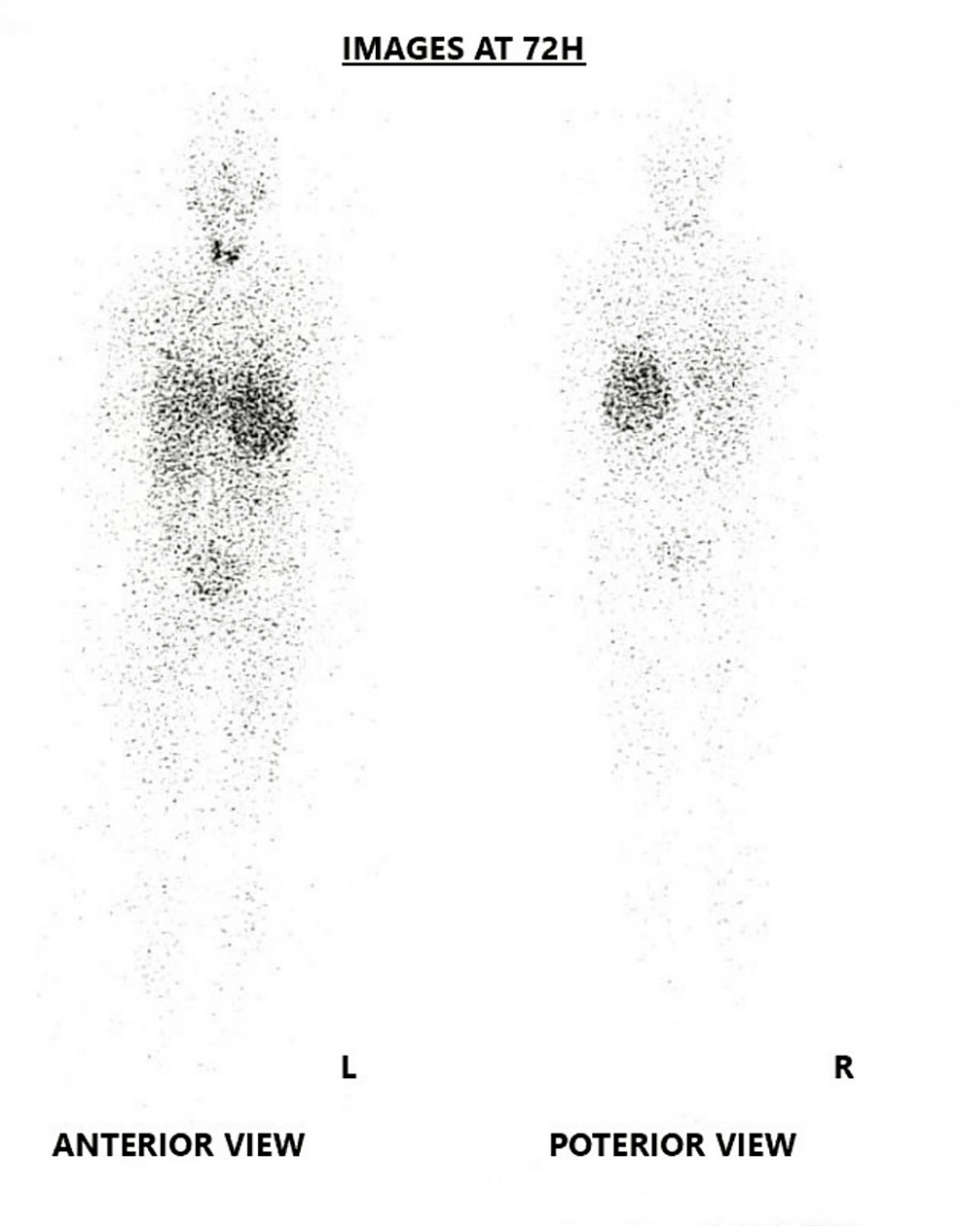
MIBG scintigraphy images showing tracer uptake of the adrenal mass. MIBG: metaiodobenzylguanidine.

CT imaging showed a 10 × 10 × 10 cm long cystic adrenal lesion, oval, with a clear outline, and a thickened wall, containing a fine contrast-enhanced septa, without signs of local invasion (Figure 4). A metaiodobenzylguanidine (MIBG) scintigraphy was performed as part of the extension workup, indicating a tracer uptake in the tumor site without any other pathological extra-adrenal uptake (Figure 5).

The diagnosis of giant cystic pheochromocytoma with neurofibromatosis type 1 was made, and the patient underwent a presurgical preparation based on alpha-blockade and non-cardio-selective beta-blockade while insisting on good hydration and education on the proscribed drugs.

After blood pressure and volemia control were obtained preoperatively, the patient underwent left adrenalectomy with monobloc removal of the adrenal tumor. Hypertensive spikes occurred intraoperatively during the manipulation and were resolved by nicardipine boluses.

**Figure 6:**
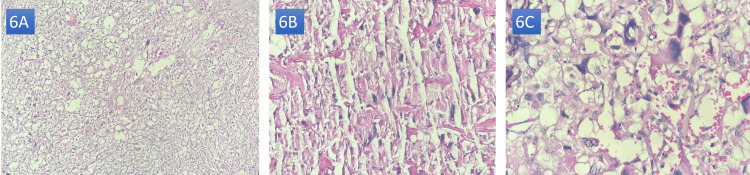
Histopathological images: H&E stains in (A) 40× view, (B) 200× view, and (C) 1000× view. Note: These images were kindly provided by Professor L. Chbani and Dr. M. Zaryouhi.

The macroscopic examination of the surgical specimen showed a soft ovoid mass of 12 × 12 cm, with the presence of multiple cystic remodeling on opening and no capsular invasion. The anatomopathological study showed adrenal parenchyma dissociated by a malignant tumoral proliferation of diffuse growth, with high cellularity, made of pleomorphic cells, clear cytoplasm, nuclear hyperchromasia, and marked atypical mitotic figures, with a high number of mitoses: 16/10 high power field (HPF), and the presence of cystic, hemorrhagic, and hemosiderin deposits (Figures 6A-6C). This was a histological appearance suggestive of a rather aggressive pheochromocytoma with a pheochromocytoma of the adrenal gland scaled (PASS) score calculated at 9.

**Figure 7:**
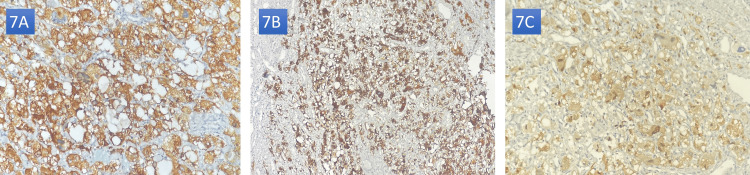
Histochemistry of the tumor with strong positivity to synaptophysin (A), chromogranin (B), and S100 (C). Note: These images were kindly provided by Professor L. Chbani and Dr. M. Zaryouhi.

Immunohistochemistry was performed for confirmation. The tumor cells were reactive of chromogranin A, synaptophysin, and S-100 confirming the diagnosis of pheochromocytoma (Figures 7A-7C).

The postoperative outcome was marked by the occurrence of high blood pressure with tachycardia during the first three days postoperatively, with spontaneous resolution after cessation of symptomatic treatment; no hypoglycemia was noted. At seven days postoperative, the urine concentration of catecholamine metabolites was at normal levels (normetanephrine: 148 nmol/d (<281), metadrenaline 39 nmol/d (<159), 3-ortho-methyl-dopa: 239 nmol/d (<329)). The patient was evaluated at one month, six months, and one-year post-surgery; her heart rate, blood pressure, and urinary catecholamines were normal under no medical treatment (Table 2).

**Table 2:** Evolution of 24 h urine catecholamines. ULN: upper limit of normal.

	Preoperative (ug/24 h)	Seven days postoperative (ug/24 h)	One month postoperative (ug/24 h)	Six months postoperative (ug/24 h)	One year postoperative (ug/24 h)	Normal values (ug/24 h)
Metanephrines	515 (16.6×ULN)	8	10	21	15	<31
Normetanéphrines	659 (12.9×ULN)	27	25	32	29	<51
3-Ortho-methyl dopa	138 (2.5×ULN)	40	36	42	44	<55

The need for lifelong follow-up was explained to the patient, given the high risk of recurrence due to her NF1 and the tumor size of the pheochromocytoma. She had also received genetic counseling; a genetic study to look for NF1 gene mutations was not necessary since the diagnosis was made clinically. The two daughters with cafe au lait spots were referred to the pediatric department for further assessment. 

## Discussion

Pheochromocytomas are catecholamine-secreting adrenal tumors that arise from chromaffin cells located in the adrenal medulla. They may occur sporadically (mainly at the fourth-fifth decade of life) or hereditarily, in which case they are part of a syndromic framework [5]: familial pheochromocytoma-paraganglioma by SDHx mutation, von Hippel-Lindau disease, multiple endocrine neoplasia type 2, and neurofibromatosis type 1, as such in the present case.

Neurofibromatosis type 1 (also known as von Recklinghausen disease) is an autosomal dominant phacomatosis characterized by pigmentary skin disorders as well as tumor development from neural crest cells. The diagnosis of NF1 remains mainly clinical and is based on the Haute Autorité de Santé (HAS) revised diagnostic criteria of 2021 [8]. This was the case for our patient, who presented more than two clinical criteria, thus allowing the diagnosis of NF1.

The association between NF1 and pheochromocytoma is well-established. Its incidence is estimated to be 0.1-5.7%, which is 10 times higher than in the general population [9]. Pheochromocytomas occurring in NF1 are characterized by germline mutations in the NF1 gene favoring the cysteine-serine-rich domain [10], making it behave like sporadic pheochromocytomas except for the increased risk of malignancy [11]. As found in different case reports in the literature, they are more likely to be unilateral, secreting mostly epinephrine, asymptomatic in 80% of cases and more than 50% of cases non-secreting, with an estimated malignancy rate of 10% [7,12,13].

One of the unique characteristics of the present case is the cystic component and the large size of the pheochromocytoma. Few cases of cystic pheochromocytomas have been reported in the literature, and even fewer of large size. They are essentially the result of cystic degeneration, extensive intra-tumoral hemorrhage, or necrosis with capsular fibrosis, which renders the interstitial tissue predominant and, therefore, biologically inactive [2,14]. The intra-capsular fluid content is rich in catecholamines and metanephrines, with a risk of a systemic passage during the manipulation of the mass and, therefore, a higher risk of hypertensive spikes per-operatively [15].

Clinically, cystic pheochromocytomas are characterized by a higher rate of asymptomatic forms, with an assay of urinary catecholamine metabolites often coming back negative [2,16]. Radiologically, they can be confused with other abdominal cystic masses [4]. They are most often unilateral [17] and are, as in the present case, larger at diagnosis than their solid counterparts [14]. The CT scan shows a peripheral contrast with a hypodense fluid content [1]. In cases of giant cystic pheochromocytomas, Imaging may not accurately define the organic site of the mass. Large adrenal tumors may also cause gland atrophy, making the residual gland unrecognizable [3]. MRI helps in identifying the nature of the cystic lesion: hemorrhage will appear as hypointense heterogeneous on T1 and T2 and necrosis hyperintense on T2 images [1]. Functional imaging will be particularly useful to differentiate a cystic pheochromocytoma from a benign adrenal cyst.

The malignant pheochromocytoma is defined by the presence of metastases in non-chromaffin tissue [3]. The risk factors for metastasis are young age at diagnosis (<40 years), extra-adrenal location, tumor size greater than 5 cm, and the presence of a mutation in one of the SDHx genes [18,19]. Some authors do not consider size as a risk factor for malignancy in view of several case reports of benign giant pheochromocytomas [2,14,20]. In the present case, she was considered to be at high risk of malignancy due to her young age at diagnosis and the tumor size of 10 cm.

Some histological prognosis scores (such as PASS, grading system for adrenal pheochromocytoma and paraganglioma (GAPP), and composite pheochromocytoma/paraganglioma prognostic (COPP) scores) have been proposed, but none of them has been agreed upon consensus [21]. Our patient had a PASS score of 9 despite a negative extension workup, justifying long-term monitoring given the high risk of metastasis.

The treatment of pheochromocytoma is surgical. Seven to 14 days of preoperative alpha-blockade with optimization of volemia is used against the cardiovascular effects of the circulating catecholamines [22]. Our patient benefited from optimal preoperative preparation, which prevented fatal complications during surgery. 

In the case of cystic pheochromocytoma diagnosed during the operative procedure, several authors agree that resection of the mass immediately, while managing intraoperative blood pressure lability, is preferable to reporting surgery, even though this may expose the patient to a higher risk of intraoperative complications [1]. An early isolation of the venous tumor drainage [23] with minimal manipulation, while avoiding rupture of the cystic components reduces the risk of hemodynamic complications and intraoperative tumoral dissemination [1]. Adrenalectomy can be performed by laparoscopic or laparotomic approach. Compared to the open approach, laparoscopic adrenalectomy is associated with less postoperative pain, shorter hospital stays, and a reduced rate of complications [22]. Laparotomy is preferred for large (>6cm) or malignant tumors [22] in order to limit the risk of tumor invasion, as it was in our case.

Pheochromocytomas have an excellent prognosis, with a five-year survival rate exceeding 95% in benign tumors and an estimated recurrence rate of 10-16% [24], which justifies long-term follow-up for at least 10 years, or even a lifetime in the case of an hereditary form; surveillance is intensified in cases of high potential risk of malignancy [22]. In our patient, the postoperative period was uneventful, with normalization of blood pressure and urinary catecholamines up to one year postoperatively.

Considering the high frequency of surgical interventions for NF1 patients, several authors recommend systematic screening for pheochromocytoma in the NF1 population every five years from the age of 35 years, based on the measurement of urinary catecholamines and eventual conventional adrenal imagery [6]. Early detection allows partial adrenalectomy, thus preserving healthy adrenal tissue; an important notion considering the risk of developing contralateral pheochromocytoma [6].

Comparison to other cases in the literature

We found 13 cases in the literature reporting cystic pheochromocytomas occurring in the setting of neurofibromatosis type 1 (Table 3); the mean age is 48 years, consistent with what was found in the literature; there was a clear female predominance with a sex ratio of 0.6. Cystic pheochromocytomas in NF1 appeared to be more symptomatic with positive urinary catecholamines compared to those occurring in a sporadic setting. They were larger in size, reaching up to 30 cm and were in the majority of cases benign (60% of cases), which is consistent with sporadic cases. Contrary to our patient, cystic pheochromocytomas are associated with gastrointestinal stromal tumors (GISTs), which are found in almost half of the reported cases (46%); these tumors develop from neural crest-derived cells, whose proliferation is regulated by neurofibromin and which expression is altered in neurofibromatosis type 1.

**Table 3:** Cases reported in the literature of cystic pheochromocytomas occurring in patients with neurofibromatosis type 1. BP: blood pressure, GAPP: grading system for adrenal pheochromocytoma and paraganglioma, GIST: gastrointestinal stromal tumor, MIBG: metaiodobenzylguanidine, NF1: neurofibromatosis type 1, PASS: pheochromocytoma of the adrenal gland scaled score.

Authors/year/country	Age/gender	Medical and family history	Clinical manifestation	Biological findings	Position/size	GIST	Malignancy	Treatment	Outcome
Lee et al., 2017, Singapore [25]	49 F	Familial history of NF1	Abdominal pain, arterial hypertension	Metanephrine elevated at 31xULN	Left adrenal gland, 18 cm	No	No	Preparation with phenoxybenzamine and atenolol. En bloc surgical resection by laparotomy	Normalization of BP and urine catecholamine levels
Goldberg et al., 2011, Canada [26]	27 F	Anxiety disorder	Headaches, palpitations, palor, arterial hypertension	Metanephrine and normetanephrine elevated at 42xULN and 30xULN	Right adrenal gland, 10 cm	No	No	Preparation with phenoxybenzamine and propranolol and nifedipine, laparotomic right adrenalectomy.	-
Yohannan et al., 2021, USA [27]	43 F	-	Incoercible vomiting, abdominal pain	Metanephrine and normetanephrine elevated at 90xULN and 30xULN	Right adrenal gland, 14 cm	No	No	Preparation with doxazosin and metyrosine, laparotomic right adrenalectomy	Normalization of urine catecholamine levels
Inkollu et al., 2022, USA [28]	60 F	Arterial hypertension	Abdominal mass	Metanephrine and normetanephrine elevated at 11xULN and 1.8xULN	Left adrenal gland, 21 cm	Yes	No	Preparation with phenoxybenzamine, laparotomic left adrenalectomy and bowel GIST resection	Normalization of BP and urine catecholamine levels
Boguslawska et al., 2022, Poland [29]	36 F	-	Adrenal incidentaloma (discovered in the context of the pheo screening in NF1)	Metanephrine and normetanephrine elevated at 35xULN and 9.8xULN	Left adrenal gland, 10 cm	No	Yes: PASS score at 10 metastasis in left kidney, liver, lung, vertebras	Preparation with doxazosin, laparotomic left adrenalectomy with nephrectomy MIBG therapy	Pancytopenia, hypercalcemia, liver cytolysis; Succumbed before second cure of MIBG therapy
Teasdale et al., 2015, Australia [30]	53 M	Atrial fibrillation, tabagism, familial history of NF1	Palpitations, sweating, vertigo, weight loss	Metanephrine and normetanephrine elevated at 15xULN and 13xULN	Right adrenal gland, 8 cm	Yes	Borderline: PASS score at 4, GAPP score at 4	Preparation with phenoxybenzamine and metoprolol, laparoscopic right adrenalectomy and bowel resection of GIST	Persistence of hypertension and plasmatic catecholamine levels
Vongsumran et al., 2020, Thailand [31]	47 M	-	Adrenal incidentaloma	Metanephrine and normetanephrine elevated at 10xULN and 5xULN	Left adrenal gland, 7 cm	No	No	Presurgical preparation, laparotomic left adrenalectomy and bowel resection of GIST	Normalization of urine catecholamine levels
Lee et al., 2020, South Korea [32]	40 F	Familial history of NF1	Adrenal incidentaloma	Metanephrine and normetanephrine elevated at 19xULN	Left adrenal gland, 6 cm	No	No	Presurgical preparation, laparotomic left adrenalectomy	Normalization of plasmatic catecholamine levels, development of ductal carcinoma of the breast
Arikan et al., 2021, Turkey [33]	54 M	Hypertension, familial history of NF1	Hypertension, abdominal mass	Metanephrine and normetanephrine elevated at 16xULN and 125xULN	Right adrenal gland, 30 cm	Yes	No	Preparation with doxazosin, laparotomic right adrenalectomy and gastric resection of GIST	Normalization of BP and urine catecholamine levels
Kramer et al., 2007, Germany [34]	63 F	Hypertension	Palpitation, hypertension, headache	Normal levels of urine catecholamines	Left adrenal gland, 5 cm	Yes	-	Presurgical preparation, laparotomic left adrenalectomy and ileal resection of GIST	Contralateral pheochromocytoma on six-month follow-up imaging
Iczkowski et al., 2018, USA [35]	54 M	Diabetes type 2, dyslipidemia, cardiopathy	Adrenal incidentaloma, hypertension	Elevated levels of urine catecholamines	Left adrenal gland, 11 cm	No	Yes: PASS score at 8, metastasis in left kidney	Presurgical preparation, laparotomic left adrenalectomy and nephrectomy	Contralateral pheochromocytoma on six-month follow-up imaging. Normal follow-up imaging
Monteiro et al., 2014, Poland [36]	50 F	Hypertension, tabagism, depression	Adrenal incidentaloma	Metanephrine and normetanephrine elevated at 8xULN and 6xULN	Right adrenal gland, 4.8 cm	No	No	Presurgical preparation, laparoscopic right adrenalectomy	Contralateral pheochromocytoma on six-month follow-up imaging
Pan et al., 2016, China [3]	56 M	Hypertension, familial history of NF1	Abdominal pain, hypertension	Normal levels of urine catecholamines	Left adrenal gland, 9 cm	Yes	No	Presurgical preparation, laparotomic left adrenalectomy and bowel resection of GIST.	Normalization of BP, normal follow-up imaging

## Conclusions

This case highlights the rarity of cystic pheochromocytomas, even more so in the context of neurofibromatosis type 1. Despite the predominant cystic component of the pheochromocytoma in the imagery of our patient, this tumor was clinically and biochemically functional, which is less frequent than what was reported in the literature. Given the high risk of cardiovascular complications, all cystic adrenal lesions should be screened for cystic pheochromocytomas. Surgery was the curative treatment for our patient, after an optimal preoperative medical preparation to decrease perioperative morbi-mortality. In this case, we also illustrate the importance of screening for pheochromocytoma in all NF1 patients, given its severity and high rate of recurrence.
